# Impact of DNA Extraction and 16S rRNA Gene Amplification Strategy on Microbiota Profiling of Faecal Samples

**DOI:** 10.3390/ijms26115226

**Published:** 2025-05-29

**Authors:** Francesca Toto, Matteo Scanu, Maurizio Gramegna, Lorenza Putignani, Federica Del Chierico

**Affiliations:** 1 Unit of Microbiome, Bambino Gesù Children’s Hospital, IRCCS, 00144 Rome, Italy; francesca.toto@opbg.net (F.T.); matteo.scanu@opbg.net (M.S.); 2 Technogenetics S.p.A., 26900 Lodi, Italy; maurizio.gramegna@technogenetics.it; 3 Unit of Microbiomics and Unit of Microbiome, Bambino Gesù Children’s Hospital, IRCCS, 00144 Rome, Italy; 4 Department of Life Science, Health, and Health Professions, Link Campus University, 00165, Rome, Italy

**Keywords:** 16S rRNA metagenomics, DNA extraction, bacterial library preparation, gut microbiota, faecal sample, PCR-based sequencing

## Abstract

High-throughput 16S rRNA metagenomic sequencing has advanced our understanding of the gut microbiome, but its reliability depends on upstream processes such as DNA extraction and bacterial library preparation. In this study, we evaluated the impact of three different DNA extraction methods (a manual method with an ad hoc-designed pre-extraction phase (PE-QIA), and two automated magnetic bead-based methods (T180H and TAT132H)) and two bacterial library preparation protocols (*home brew* and VeriFi) on the 16S rRNA-based metagenomic profiling of faecal samples. T180H and TAT132H produced significantly higher DNA concentrations than PE-QIA, whereas TAT132H yielded DNA of lower purity compared to the others. In the taxonomic analysis, PE-QIA provided a balanced recovery of Gram-positive and Gram-negative bacteria, TAT132H was enriched in Gram-positive taxa, and T180H was enriched in Gram-negative taxa. An analysis of Microbial Community Standard (MOCK) samples showed that PE-QIA and T180H were more accurate than TAT132H. Finally, the VeriFi method yielded higher amplicon concentrations and sequence counts than the *home brew* protocol, despite the high level of chimeras. In conclusion, a robust performance in terms of DNA yield, purity, and taxonomic representation was obtained by PE-QIA and T180H. Furthermore, it was found that the impact of PCR-based steps on gut microbiota profiling can be minimized by an accurate bioinformatic pipeline.

## 1. Introduction

The human gut microbiota harbours highly complex and diverse microbial communities, including bacteria, fungi, viruses, and protozoa [1]. The field of metagenomics, which uses high-throughput sequencing technologies to analyse microbial communities, faces several challenges due to the variability of the existing experimental and computational methods. Many studies have shown how different experimental processing pipelines affect the results [2,3,4,5,6,7]. In particular, the choice of DNA extraction method significantly influences the results of microbiota profiling, introducing biases that can affect the interpretation of microbial community structures and their diversity [8,9,10,11]. Indeed, wet pipelines can significantly affect the accuracy, reproducibility, and depth of microbial community analysis [12]. In particular, different DNA extraction methods have different efficiencies in recovering DNA from different microbial communities [12]. Some methods may favour the extraction of DNA from some bacterial groups to the detriment of others (e.g., Gram-positive vs. Gram-negative bacteria, or difficult-to-lyse organisms such as *Bifidobacterium* and *Lactobacillus*) [13,14]. This difference is due to structural variations in the cell wall. Specifically, the cell wall of Gram-positive bacteria contains only a lipid plasma membrane and a thick layer of peptidoglycan bound to theicoic and lipoteichoic acids, whereas Gram-negative bacteria have a thin layer of peptidoglycan in their periplasmic space and an inner and outer cell membrane, of which the latter is surrounded by a layer of lipopolysaccharide [15]. The different resistances of the two barriers to disruption can lead to a bias in the representation of the microbiota in the final profiling. In addition, the quality of the extracted DNA is critical for downstream PCR amplification, a fundamental step in bacterial library construction. Poor quality DNA samples can result in poor amplification of the 16S rRNA gene, leading to an incomplete or inaccurate representation of the microbiota in the sample. Finally, another aspect to consider is the matrix effect of the sample, whether it is biological or environmental, as it can interfere with DNA extraction, both in terms of performance and quality, affecting the overall microbial community structure and composition of the sample [16].

In addition, library preparation steps can introduce biases that affect the diversity and abundance of the bacterial taxa that are detected [17]. Variations in the amplification conditions (e.g., cycle number, temperature, polymerase Taq performance) can affect the coverage of different bacterial groups, thereby affecting the sequencing depth (number of reads per sample) [18]. Indeed, a low sequencing depth may fail to capture rare taxa, ultimately leading to a biased representation of the microbiome. In addition, a significant source of artefacts is the PCR amplification step. In fact, when the 16S rRNA genes of several species are amplified simultaneously, several stochastic mechanisms, such as polymerase dissociation, nucleotide misincorporation, and secondary structure formation, can lead to the formation of chimeric sequences [19,20]. In addition, incomplete PCR products that serve as primers for the amplification of related fragments are the most common pathway for the generation of chimeras [21]. These chimeric, or recombinant, sequences, being composed of segments from different 16S rRNA genes, can artificially inflate the sample’s diversity by introducing novel sequences that were not present in the original sample. Consequently, these artefacts can lead to an overestimation of diversity and the erroneous detection of non-existent microorganisms in microbial composition analysis [22].

Taken together, the cumulative effects of the DNA extraction efficiency, PCR amplification conditions, and library preparation protocols significantly influence the community structure, ecology, and composition that are observed in gut microbiota profiling.

In this study, we analysed the effects of three different DNA extraction methods and two bacterial library preparation protocols on the outcomes of gut microbiota profiling using 16S rRNA-based metagenomics. The effects of the experimental factors were evaluated by comparing the DNA extraction yields, sequencing performance, bacterial richness, and taxonomic diversity of the resulting profiles.

## 2. Results

### 2.1. Differences in DNA Concentration and Purity

We compared the DNA concentration and purity amongst the three DNA extraction methods. The PE-QIA was a manual method that was specific to faecal samples and was optimised by adding a thermal and mechanical bacterial wall lysis step, as described in the Section 4. The T180H was an automated stool-specific method that included a chemical pre-treatment step. The TAT132H was also an automated method which was applicable to different biological samples and included an enzymatic pre-treatment step.

**Figure 1:**
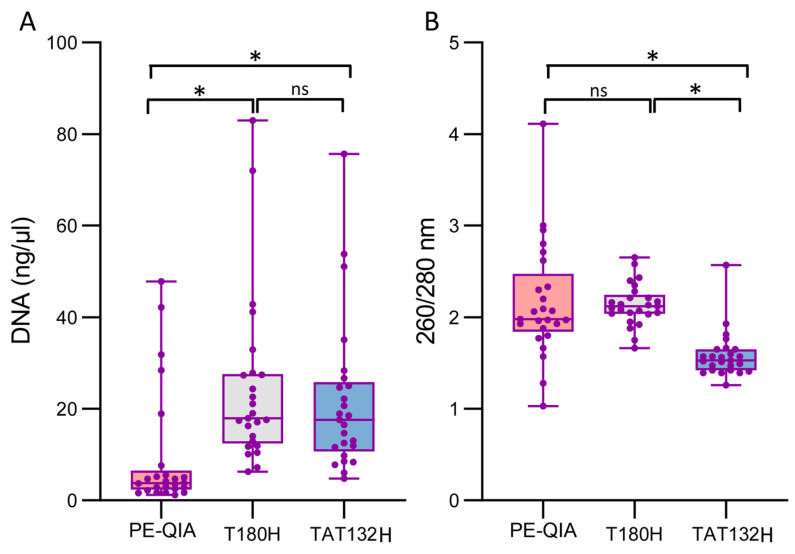
DNA yield and purity extracted from faecal samples using the three kits. DNA was extracted from 200 mg of faeces per sample (n = 25 samples per kit). Median values are indicated by the line in the box plot. The box extends from the 25th to the 75th percentile and whiskers indicate the minimum and maximum values. *t*-test was used to calculate *p*-values (* *p*-value < 0.01); (**A**) box plot showing DNA yields obtained for all kits evaluated, (**B**) plot showing absorbance ratios at 260/280 for all kits evaluated.

The samples showed a significantly higher DNA concentration when processed with the T180H and TAT132H kits compared to PE-QIA (mean ± SD: PE-QIA: 9.53 ± 13.31 ng/μL, T180H: 24.49 ± 18.60 ng/μL, TAT132H: 21.75 ± 16.78 ng/μL, paired *t*-test: PE-QIAxT180H *p*-value = 0.002, T180HxTAT132H *p*-value = 0.59, PE-QIAxTAT132H *p*-value = 0.006) (Figure 1A, Table 1).

The absorbance ratio at 260/280 nm assesses the purity of the DNA. In general, a ratio of ~1.8 is considered optimal for DNA purity; a lower ratio may indicate the presence of contaminants such as proteins, phenols, or other contaminants that absorb at around 280 nm.

The 260/280 absorbance values were significantly different for PE-QIA and T180H, showing a ratio closer to 2, while the TAT132H samples showed a lower DNA purity (mean ± SD: PE-QIA: 2.16 ± 0.63, T180H: 2.14 ± 0.23, TAT132H 1.58 ± 0.25, paired *t*-test: PE-QIAxT180H *p*-value = 0.87, T180HxTAT132H *p*-value = 1.5 × 10^−10^, PE-QIAxTAT132H *p*-value = 0.0001) (Figure 1B, Table 1).

### 2.2. Evaluation of the DNA Extraction Methods on Microbial Community Diversity

Before analysing the results of this study, we evaluated the sequencing results of the negative DNA extraction controls in order to monitor for any potential biases. A small number of raw reads were generated when sequencing all negative controls. Specifically, we obtained 371 sequences using the PE-QIA kit, 697 using the T180H kit, and 171 using the T132H kit. Almost all of these were eliminated by the bioinformatic pipeline, as they were recognised as chimeric sequences. This resulted in 0 ASVs for the PE-QIA kit, 3 ASVs for the T180H kit, and 25 ASVs for the T132H kit.

**Figure 2:**
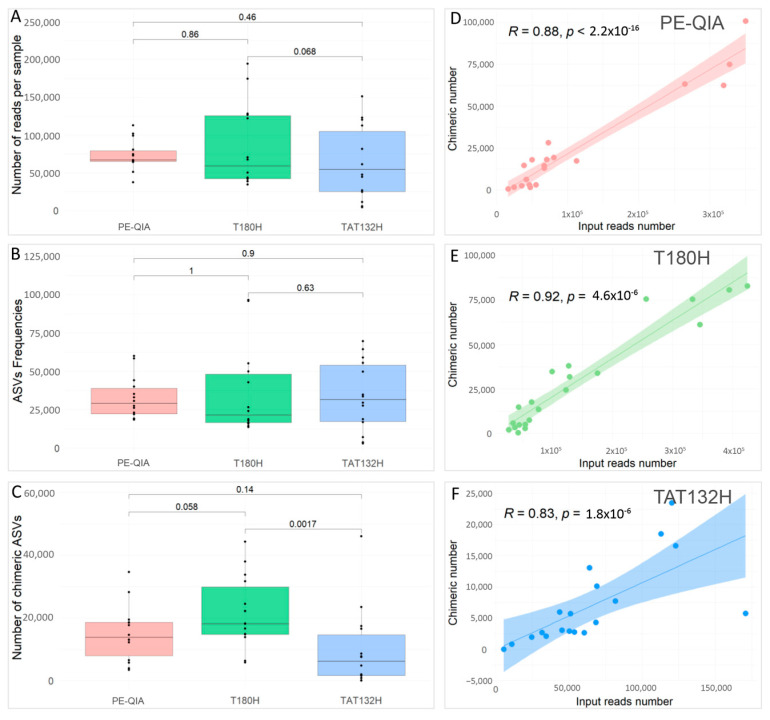
Sequencing parameters of faecal samples subjected to the three different DNA extraction methods. The box plots represent the median values and the 25th and the 75th percentiles. Wilcoxon rank test was used to calculate *p*-values. (**A**) Box plot of the number of reads per sample obtained for the three kits evaluated. (**B**) Box plot of the ASV frequencies for the three kits evaluated; (**C**) Box plot of the number of chimeras obtained for the three kits evaluated. The fitted line plots of chimeric number and input reads number represent the correlation between these two variables for the PE-QIA method (**D**), T180H method (**E**), and TAT132H method (**F**). Each sample is represented by a dot.

To compare the effects of the three different DNA extraction methods on the sequencing performance, we evaluated the number of reads per sample (Figure 2A), the ASV frequencies (Figure 2B), and the number of chimeras (Figure 2C) for each sample, grouped by DNA extraction method.

All methods gave similar results; in fact, the comparisons were not statistically significant for any variable.

However, the PE-QIA method produced the highest median number of sequences, while TAT132H produced the highest median number of ASV frequencies and the T180H had the highest median number of chimeras. Moreover, our analysis revealed a significant positive correlation between the number of chimera sequences and the number of reads per sample processed using the PE-QIA (R = 0.88, *p*-value < 0.05) (Figure 2D), T180H (R = 0.92, *p*-value < 0.05) (Figure 2E), and TAT132H (R = 0.83, *p*-value < 0.05) (Figure 2F) methods.

**Figure 3:**
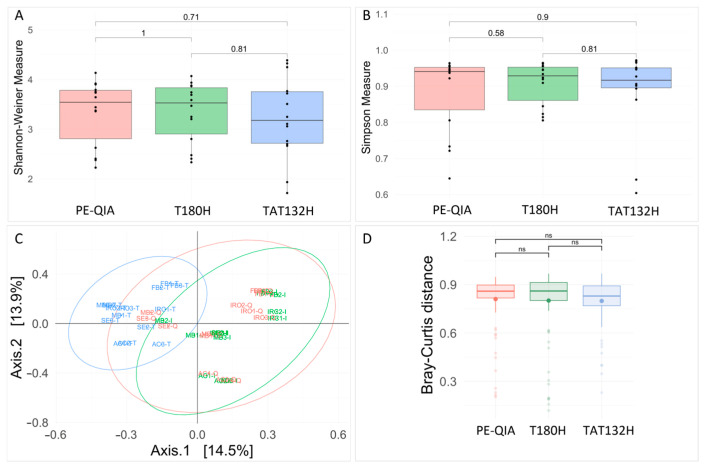
Analysis of alpha and beta diversity. Alpha diversity is based on the Shannon (**A**) and Simpson (**B**) indices. Pairwise Wilcoxon rank test for comparison of DNA extraction methods shows no statistical significance. Beta diversity was calculated using the Bray–Curtis distance and represented by PCoA plot (**C**) and box plot (**D**). PERMANOVA test is statistically significant, *p*-value = 0.001, R^2^ = 0.141.

Richness analysis showed no significant differences amongst the three methods for both the Shannon and Simpson indices (Figure 3A,B). However, both the Shannon and Simpson diversity indices showed lower diversities for the TAT132H method. All diversity values are reported in Supplementary Table S1. We assessed the differences in the observed microbial communities among the samples that were extracted using each protocol. A PCoA plot based on Bray–Curtis distances showed the clear clustering of samples extracted by the TAT132H method (Figure 3C,D). PERMANOVA analysis confirmed the significant effect of the DNA extraction protocol on the resulting microbial community composition (*p*-value = 0.001).

To test how many and which bacterial taxa were extracted by all kits and which were uniquely extracted, we constructed a Venn diagram.

**Figure 4:**
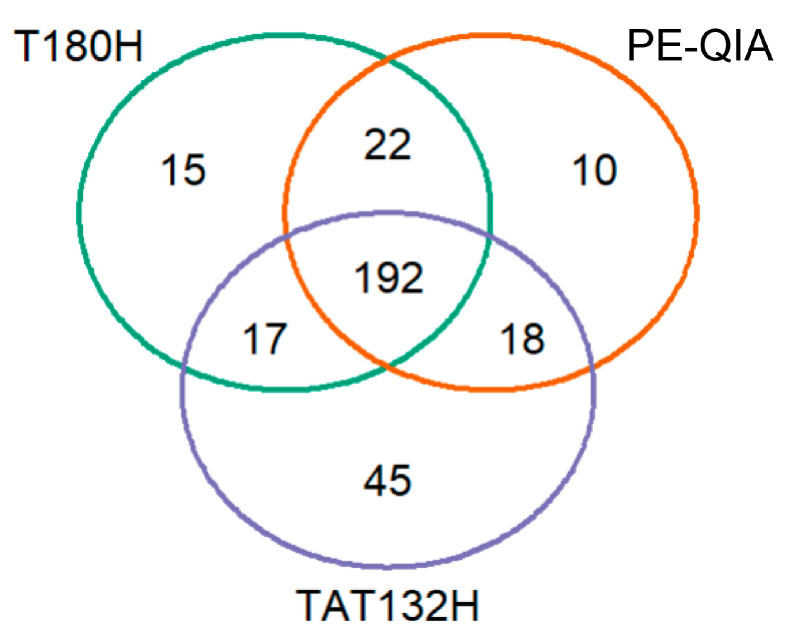
Venn diagram of bacterial taxa shared across DNA extraction methods.

The diagram revealed a total of 192 bacterial taxa across all sample groups. Specifically, 22 taxa were detected exclusively by both the PE-QIA and T180H methods, 18 exclusively by PE-QIA and TAT132H, and 17 exclusively by TAT132H and T180H. Additionally, 45 taxa were uniquely identified by TAT132H, 15 by T180H, and 10 by PE-QIA (Figure 4).

Interestingly, among the 45 unique bacterial taxa that were present in the samples treated with the TAT132H method, numerous genera belonging to the Firmicutes_D (e.g., *Bulleidia*, *Faecalicoccus*, *Lacticaseibacillus*, *Leuconostoc*_B, *Limosilactobacillus*, *Lactococcus* _A_343306, *Enterococcus*) and to Actinobacteriota (e.g., *Rothia*, *Propionibacterium*, *Adlercreutzia*_404257, *Corynebacterium*, and *Mobiluncus*) phyla were present.

Among the unique taxa identified in the T180H samples were numerous genera belonging to the Proteobacteria phylum (e.g., *Agrobacterium*, *Phyllobacterium*_498581, *Pseudomonas*, and *Sphingomonas*_L_486704). Finally, the unique taxa identified in the PE-QIA samples included genera belonging to both the Actinobacteriota and Proteobacteria phyla.

### 2.3. Evaluation of the DNA Extraction Methods on Microbial Community Composition

**Figure 5:**
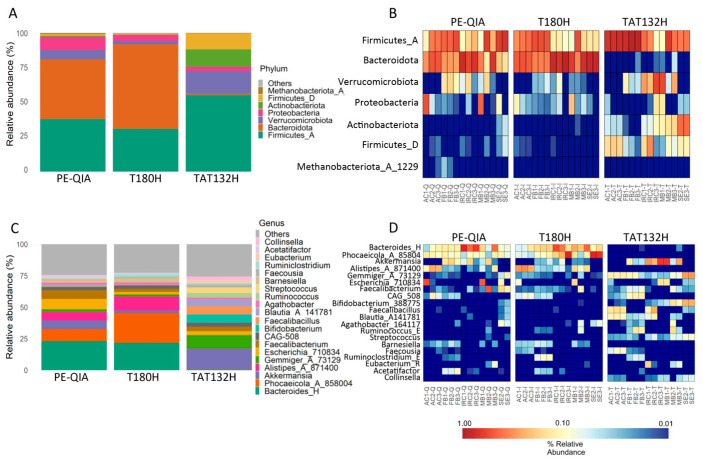
Method-dependent variations in the relative abundance of microbiota profiles at the phylum (**A**,**B**) and genus (**C**,**D**) levels. (**A**,**C**) Histograms of the relative abundances of the 8 most abundant phyla and 20 most abundant genera obtained by the sequencing of the faecal samples treated with three different DNA extraction methods. (**B**,**D**) Heatmap showing, for each sample, the relative abundance of the 8 most abundant phyla and 20 most abundant genera.

We evaluated the relative abundance of microbial taxa at the phylum and genus taxonomic levels. The analysis of gut microbiota profiles at the phylum level showed a similarity between the results of the PE-QIA and T180H methods. However, the results of both methods differed from those of the TAT132H method (Figure 5A). Surprisingly, TAT132H was enriched in Firmicutes_D, Actinobacteriota, Verrucomicrobiota, and Firmicutes_A, markedly reduced in Bacteroidota, and somewhat reduced in Proteobacteria compared to the other two methods (Figure 5B).

At the genus level, we showed that PE-QIA samples were enriched in *Bacteroides*_H, *Phocaeicola*_A_858004, *Akkermansia*, *Alistipes*_A_871400, *Escherichia*_710834, and *Faecalibacterium*. The T180H samples were enriched in *Bacteroides*_H, *Phocaeicola*_A_858004, and *Alistipes*_A_871400. Otherwise, the TAT132H samples were enriched in *Akkermansia*, *Gemmiger*_A_73129, *Escherichia*_710834, *Faecalibacterium*, *Bifidobacterium*_388775, *Faecalibacillus*, *Blautia*_A_141781, and *Streptococcus* (Figure 5C,D).

**Figure 6:**
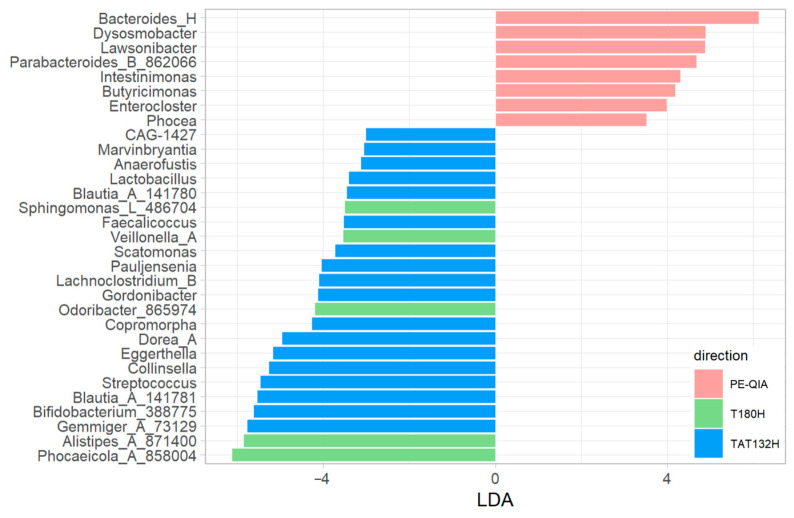
Comparative analysis at genus level between DNA extraction methods used on faecal samples. Linear discriminant analysis effect size (LEfSe) was calculated at genus level. The *p*-values were FDR-adjusted (*p*-value < 0.05) and LDA score was >3.

The comparison of the relative abundances between the three groups of samples, calculated with univariate analysis LEfSe, highlighted differences in the relative abundances of some genera (Figure 6).

In particular, Bacteroides_H, Lawsonibacter, Dysosmobacter, Enterocloster, Butyricimonas, Parabacteroides_B_862066, Intestinimonas, and Phocea were significantly increased in the PE-QIA samples; Phocaeicola_A_858004, Alistipes_A_871400, Sphingomonas_L_486704, Odoribacter_865974, and Veillonella_A were significantly increased in T180H; and, finally, Pauljensenia, Collinsella, Streptococcus, Scatomonas, Eggerthella, Gemmiger_A_73129, Bifidobacterium_388775, Dorea_A, CAG-1427, Lactobacillus, Blautia_A_141781, Anaerofustis, Gordonibacter, Blautia_A_141780, Lachnoclostridium_B, Copromorpha, Faecalicoccus, and Marvinbryantia were significantly increased in TAT132H.

### 2.4. Evaluation of the DNA Extraction Methods on Bacterial Wall Cell

By selecting genera that were statistically significant according to the LEfSe analysis and classifying them according to their Gram classification, we showed that the T180H was enriched in Gram-negative bacteria, the TAT132H group was enriched in Gram-positive bacteria, and the PE-QIA was enriched in both Gram groups (Table 2). This agreed with the previous data on the unique taxa associated with each extraction method.

### 2.5. Evaluation of DNA Extraction Methods on the Microbial Community Standard (MOCK) Community

**Figure 7:**
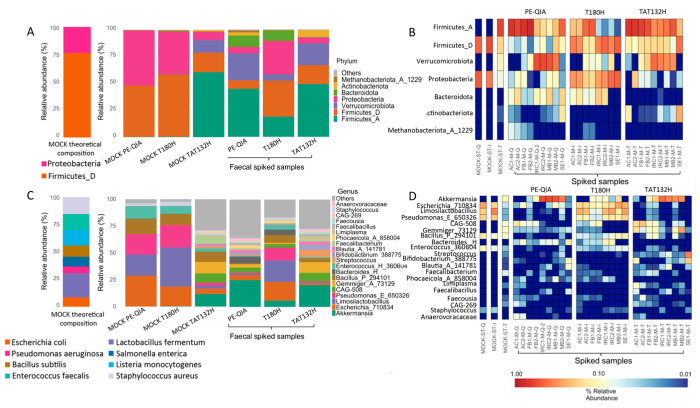
Theoretical and actual relative abundances of MOCK and spiked samples at phylum (**A**,**B**) and genus (**C**,**D**) levels. (**A**,**C**) Histograms of the relative abundances of the 7 most abundant phyla and 20 most abundant genera obtained by the three different DNA extraction methods. (**B**,**D**) Heatmap showing, for each samples, the relative abundances of the 7 most abundant phyla and 20 most abundant genera.

To evaluate the accuracy of the three DNA extraction methods, a commercial Microbial Community Standard (MOCK) sample and samples spiked with MOCK were treated with the three DNA extraction methods (Figure 7). At the phylum level, the MOCK samples extracted with PE-QIA and T180H were similar in composition to the theoretical composition of MOCK, with the relative abundance proportions of Proteobacteria and Firmicutes_D being inverted. The MOCK sample treated with TAT132H showed a completely different profile from the theoretical one. In fact, Firmicutes_A, Verrucomicrobia, and Actinobacteria were present, which were not present in the theoretical MOCK composition (Figure 7A,B).

At the genus level, the MOCK samples extracted with PE-QIA and T180H showed the same composition, with the presence of *Escherichia*_710834, *Limosilactobacillus* (ex. *Lactobacillus fermentum*, [23]), *Pseudomonas*_E_650326, *Bacillus*_P_294101, *Enterococcus*_H_360604, and *Staphylococcus*, mimicking the theoretical microbial community better than the TAT132H method, although neither accurately reproduced the reported relative abundance of each taxa in terms of quantity. We also observed an underrepresentation of *Salmonella enterica* and *Listeria monocytogenes* in all samples. The profile of the MOCK sample treated with TAT132H was characterised by the presence of, among others, *Akkermansia*, *Escherichia*_710834, *Limosilactobacillus*, *Pseudomonas*_E_650326, CAG-508, *Gemmiger*, *Bacillus*_P_294101, *Enterococcus*_H_360604, and *Faecalibacterium*. Finally, the spiked samples treated with T180H showed an intermediate composition between the MOCK and faecal samples with respect to PE-QIA and TAT132H (Figure 7C,D), indicating a more accurate extraction step.

The selection of genera based on the theoretical composition of the MOCK showed that TAT132H had the lowest performance compared to the other two DNA extraction kits (Table 3).

In both the spiked and MOCK samples treated by all three kits, a marked underrepresentation of *Listeria* and *Salmonella* was evident. The best performance was achieved by T180H in the spiked samples and by PE-QIA in the MOCK samples. However, both kits showed a lower representation of *Staphylococcus* in both types of samples compared to the theoretical composition declared by the manufacturer of the MOCK standard. *Bacillus*_P_294101, *Enterococcus*_H_360604, *Escherichia*_710834, *Pseudomonas*_E_650326, and *Limosilactobacillus* were overrepresented in the MOCK samples.

### 2.6. Evaluation of the Effects of Bacterial Library Amplification Protocol on Microbial Community Composition

We evaluated the performance of two different PCR amplification protocols, *home brew* and VeriFi, on the studied gut microbiota profiling procedures.

**Figure 8:**
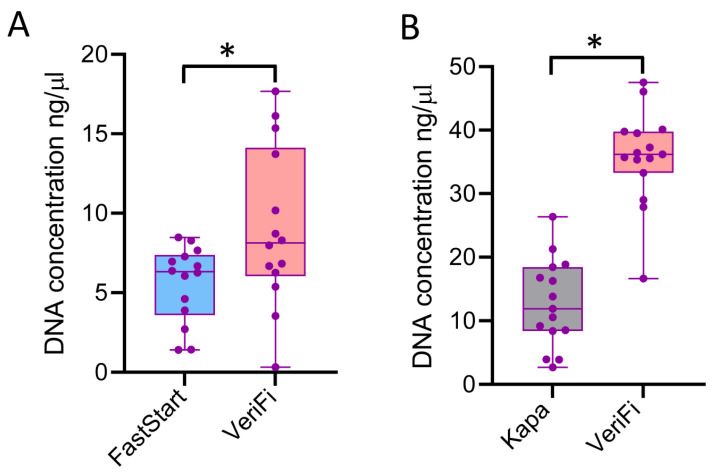
DNA concentration of amplicons obtained during bacterial library preparations with two protocols, *home brew* and VeriFi. Amplicons were quantified by Nano Drop^®^ ND-1000. Median values are indicated by the line in the box plot. The box extends from the 25th to the 75th percentile and whiskers indicate the minimum and maximum values. *t*-test was used to calculate *p*-values (*p*-value *); (**A**) box plot showing DNA concentrations obtained during the amplification of the 16S rRNA V3–V4 regions, *p*-value = 0.026; (**B**) box plot showing DNA concentrations obtained during the barcoding step using Nextera^TM^ primers, *p*-value = 1.6 × 10^−9^.

In gut microbiota profiling, the first step consists of bacterial library construction based on the amplification of the V3–V4 regions of the 16s rRNA gene. In this step, we compared the performance of the Fast Start kit (Fast Start Hifi Taq, Roche Diagnostics, Indianapolis, Indiana, USA) and the VeriFi kit (PCR Biosystems simplifying research), showing a significant increase in the DNA concentration of amplicons that was obtained with the VeriFi kit (mean ± SD: Fast Start: 5.57 ± 2.39 ng/μL, VeriFi: 9.07 ± 5.02 ng/μL, paired t-test: *p*-value = 0.026) (Figure 8A).

The second step in the bacterial library construction was PCR indexing. In this step, we compared the performance of the Kapa kit (KAPA Biosystems Inc., Wilmington, MA, USA) with that of the VeriFi kit (PCR Biosystems simplifying research), showing a significant increase in the DNA concentration of the amplicons obtained with the VeriFi kit (mean ± SD: Kapa: 12.72 ± 6.91 ng/μL, VeriFi: 35.75 ± 7.45 ng/μL, paired *t*-test: *p*-value = 1.6 × 10^−9^) (Figure 8B).

**Figure 9:**
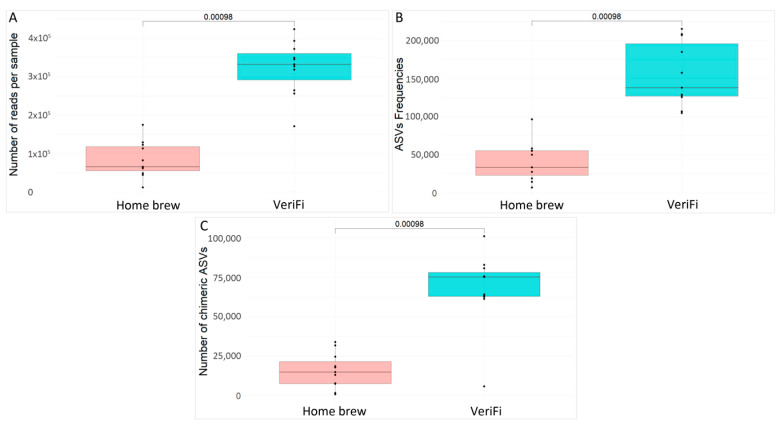
Sequencing parameters of amplicons obtained using the two protocols of amplification, *home brew* and VeriFi. The box plots report the median value and the 25th and the 75th percentiles. Wilcoxon rank test was used to calculate *p*-values. (**A**) Box plot showing the number of reads per sample obtained for the three kits evaluated. (**B**) Figure showing the ASV frequencies for the three kits evaluated. (**C**) Box plot showing the number of chimeras obtained for the three kits evaluated.

We also evaluated the number of reads per sample (Figure 9A), the ASV frequencies (Figure 9B), and the number of chimeras (Figure 9C) obtained for each protocol.

The VeriFi protocol produced a higher statistically significant number of sequences, a higher number of ASV frequencies, and, finally, the number of chimeras.

**Figure 10:**
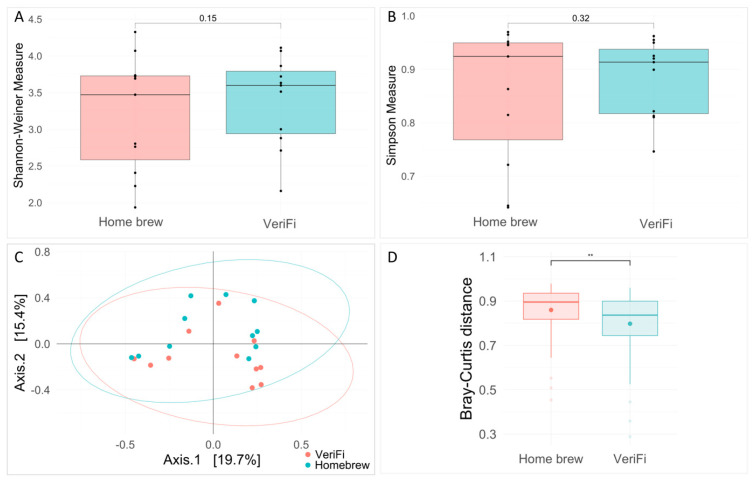
Analysis of alpha and beta diversity. Alpha diversity was based on the Shannon (**A**) and Simpson (**B**) indices. The pairwise Wilcoxon rank test, used to compare the two protocols of amplification, *home brew* and VeriFi, indicated no statistically significant differences. Beta diversity was calculated using the Bray–Curtis distance and represented by PCoA plot (**C**). PERMANOVA test is statistically significant, *p*-value = 0.035, R^2^ = 0.082. The Bray–Curtis distance was represented by box plot (**D**). **, *p* value < 0.001.

Ecological analysis showed no significant differences between the two protocols for both the Shannon and Simpson indices (Figure 10A,B). Bray–Curtis distance analysis showed a clustering of the samples that were grouped for the library construction protocols (PERMANOVA, *p*-value = 0.035) and a statistically significant reduction in distance for the VeriFi samples (Figure 10C).

**Figure 11:**
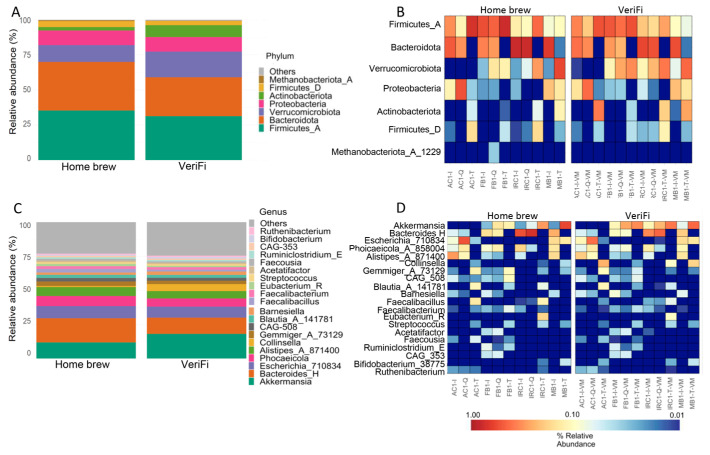
Protocol-dependent variations in the relative abundance of microbiota profiles at the phylum (**A**,**B**) and genus (**C**,**D**) levels. (**A**,**C**) Histograms of the relative abundances of the 7 most abundant phyla and 20 most abundant genera obtained by the sequencing of amplicons obtained during bacterial library preparations with two protocols, *home brew* and VeriFi. (**B**,**D**) Heatmap showing, for each sample, the relative abundance of the 7 most abundant phyla and 20 most abundant genera for each sample.

The distribution of the relative abundance of microbial taxa at the phylum level showed an increase in Firmicutes_A, Bacteroidota, and Firmicutes_D and a reduction in Verrucomicrobia and Actinobacteriota in the *home brew* protocol compared to the VeriFi protocol (Figure 11A,B). Surprisingly, the levels of Proteobacteria were similar in both protocols (Figure 11A,B).

At the genus level, we showed that the home brew samples were enriched in *Bacteroides*_H, *Phocaeicola*_A_858004, and *Alistipes*_A_871400, while *Akkermansia* and *Collinsella* were reduced (Figure 11C,D). As reported for Proteobacteria, the relative abundance of *Escherichia*_710834 was similar for both protocols (Figure 11C,D).

## 3. Discussion

The human gut microbiota is a complex ecosystem that harbours diverse microbial communities shaped by a variety of intrinsic and extrinsic factors. Reconstructing the exact map of its inhabitants is therefore an ongoing challenge. In fact, there are several factors that can influence the results of gut microbiota profiling, including the composition of the microbial community, the cell wall structure of the microorganisms, the nature of the biological samples, and the physiological state of the host. These factors, together with the use of different wet-lab protocols (e.g., DNA extraction methods and library preparation protocols), can then differently capture and represent microbial content, introducing bias into gut microbiota profiling [24,25,26]. Several studies have shown that the use of different commercial DNA extraction kits significantly influences the sequencing results of faecal samples [27,28,29,30,31].

In this study, we compared three extraction methods to identify the approach that most accurately recovers DNA from faecal samples. Our results indicate that both the T180H and TAT132H extraction methods yielded higher DNA concentrations compared to PE-QIA, although the TAT132H method yielded the lowest DNA purity value compared to the other two methods.

In general, the column-based DNA extraction kits based on affinity technology perform better than the magnetic bead-based kits, providing better purification and yielding DNA with fewer contaminants [32,33]. This is particularly important for faecal samples, which are inherently complex and may contain inhibitors that interfere with downstream PCR procedures.

The bead-based DNA extraction methods are based on the binding of DNA to a positively charged magnetic bead at pH ≤ 6.5 and the release of DNA at pH ≥ 8.5 [33,34]. Although these methods are less efficient in terms of purity, they are faster and more automated protocols that reduce handling errors [33].

To estimate the performance of each DNA kit, we evaluated the metagenomic outcomes and downstream sequencing performance of each kit using several statistical analyses. The PE-QIA method exhibited the best overall performance, yielding the highest median sequence counts, a greater number of distinct ASVs, and the fewest chimeras. In contrast, the T180H method produced the highest median chimera count. Notably, after applying an ad hoc bioinformatic workflow to mitigate chimera bias, the samples processed with PE-QIA and T180H exhibited a high level of similarity in their beta diversity as well as in their phylum and genus profiles. Additionally, the TAT132H method yielded the highest median ASV frequencies. Although the median number of reads obtained by sequencing was comparable between the samples when they were categorised by protocol, different correlation coefficients emerged between this quantity and the number of chimeras across these protocols. The observed positive correlation suggests that the substantial number of reads obtained by sequencing may facilitate chimera formation, possibly due to artefacts introduced during the amplification or sequencing procedures. Notably, this analysis showed a slight increase in the correlation between these two variables for the T180H protocol compared to the other two, suggesting that this protocol may be particularly susceptible to chimera formation.

Overall, these results highlight the importance of implementing rigorous bioinformatic filtering strategies to detect and remove such anomalies, and to thereby ensure an improved data quality and more reliable downstream analyses [35].

In addition, alpha diversity analysis revealed that the TAT132H method had lower ASV richness compared to the other methods. This reduced richness, combined with the increased ASV frequencies, suggests an overrepresentation of certain ASVs, indicating a potential sequencing bias.

The Venn diagram showed the consistency of the sequencing results of all methods for 192 out of a total of 319 genera (61%). However, TAT132H showed the highest number of uniquely identified genera, most of which belonged to the Firmicutes_D and Actinobacteriota phyla. PE-QIA and T180H showed 22 common genera, highlighting the similarity in the results of these two methods, although the T180H group was enriched in unique genera belonging to Proteobacteria and the PE-QIA group was enriched in unique genera belonging to both Actinobacteriota and Proteobacteria.

The Firmicutes/Bacteroidota (F/B) ratio was different between the three sample groups (F/B PE-QIA= 0.91; F/B T180H= 0.52 and F/B TAT132H = 65.57), showing the marked underrepresentation of Bacteroidota in TAT132H. However, in contrast to the other two, TAT132H was enriched of Verrucomicrobiota, Actinobacteroidota, and Firmicutes_D. These differences were also highlighted at the genus level with the enrichment of, among others, *Bacteroides*_H and *Parabacteroides*_B_862066 (Bacteroidota) in the PE-QIA samples; Phocaeicola_A_858004, Alistipes_A_871400 (Bacteroidota), and *Veillonella*_A (Firmicutes) in T180H; and, finally, Blautia_A_141781, Dorea_A, Gemmiger_A_73129 (Firmicutes_A), *Collinsella*, *Eggerthella*, Bifidobacterium_388775 (Actinobacteriota), and *Lactobacillus* (Firmicutes_D) in TAT132H.

Interestingly, when the bacteria were grouped according to their Gram staining, the emerging results indicated the selectivity of TAT132H for DNA extraction from Gram-positive and T180H from Gram-negative samples, while PE-QIA showed the best performance for both Gram-positive and -negative samples.

The difference in recovery between Gram-positive and -negative samples may be due to the pre-treatment steps. Improving the lysis step in DNA extraction for metagenomics is critical because efficient cell disruption is essential to obtaining high quality DNA from a wide range of microorganisms from different sample matrices. The PE-QIA method, with the addition of mechanical lysis by bead-beating at high temperatures and freeze–thaw cycles, results in improved cell wall disruption and the improved release of DNA from difficult-to-lyse microorganisms such as Gram-positive bacteria. The T180H and TAT132H methods offer a pre-treatment step of chemical/enzymatic lysis with an undefined pre-treatment solution (T180H) or a lysozyme solution (TAT132H) combined with a high incubation temperature, but this may not be sufficient for tougher cells. Lysozyme, an alkaline glucosidase enzyme, hydrolyses bacterial polysaccharides and degrades peptidoglycan, resulting in cell lysis [36]. However, Gram-negative bacterial strains are usually resistant to natural lysozyme because they have a thin layer of peptidoglycan and an outer membrane that can prevent antibacterial substances such as lysozyme from entering the bacteria [36]. It is also recommended to increase the thermal lysis temperature from 50 °C to 95 °C [37] and to include bead-beating [9] to improve the recovery of Gram-positive DNA.

The performance of the three DNA extraction methods was further evaluated using a commercial MOCK sample. This MOCK community, which contains known gut taxa in predetermined proportions and includes both Gram-positive and Gram-negative bacteria (as detailed in the Section 4), served as a benchmark to validate and compare the methods [38]. The results confirmed that the PE-QIA and T180H methods produced profiles that closely matched the theoretical composition of the MOCK community. In contrast, the TAT132H method produced a profile that was markedly different, although it was similar to that of a stool sample. This could be due to cross-contamination resulting from DNA carryover from previous samples, despite measures having been implemented to prevent such issues. Even when this result was excluded, substantial evidence was obtained to suggest that the TAT132H kit performed poorly: a lower DNA purity, a lower diversity index values, and divergent microbiota profiles between the stool and MOCK-spiked samples compared to samples treated with the other two kits. For these reasons, we are convinced that the TAT132H kit performs worse than the other two, regardless of the contamination event.

Finally, we evaluated the performance of two different PCR protocols in the gut microbiota profiling workflow. The use of the VeriFi kit in both library construction and PCR indexing showed the production of a high concentration of amplicons. This result was confirmed by analysis of the resulting sequencing performance. It was evident that the VeriFi protocol yielded a higher number of sequences and ASV frequencies compared to the *home brew* method. The VeriFi Library Amplification Mix contains an aptamer that imparts greater hot start activity to the polymerase than the *home brew* using antibodies, which results in a greater ability to bind DNA due to inhibiting the 3′–5′ exonuclease and 5′–3′ polymerase activities of the enzyme at temperatures below 40 °C. However, this new amplification method also produced a higher number of chimeras than the *home brew* method, showing a low sequencing accuracy. Nevertheless, only small differences in the composition of the gut microbiota were observed between the two methods. In particular, in the VeriFi group, we showed a decrease in *Bacteroides*_H, *Phocaeicola*_A_858004, and *Alistipes*_A_871400 and an increase in *Akkermansia* and *Collinsella* compared to the *home brew* method. These results highlighted that DNA extraction methods are a major driver of microbiota profiling with respect to downstream phases. Indeed, the production of large numbers of chimeras during PCR can be corrected by a robust and complete bioinformatics pipeline, whereas there is no way to correct for differences resulting from DNA extraction from some bacteria and not others.

## 4. Materials and Methods

### 4.1. Sample Description and Handling

This project was approved by the Ethical Committee of the Bambino Gesù Children’s Hospital (OPBG) of Rome, Italy, with the approval number 2853_OPBG_2022. Then 16 faecal samples used in this study were provided by the OPBG’s Microbiome Unit sample collection.

### 4.2. Preparation of Faecal Sample Artificially Spiked with MOCK Community

The accuracy and bias of each DNA extraction method was assessed using a commercially available MOCK community (ZymoBIOMICS Microbial Community Standard, Catalogue no. D6300, Zymo Research Corp., Irvine, CA, USA). This mixed microbial community consists of 8 bacteria, 5 Gram-positive (*Bacillus subtilis*, *Listeria monocytogenes*, *Staphylococcus aureus*, *Enterococccus faecalis*, and *Lactobacillus fermentum*) and 3 Gram-negative (*Salmonella enterica*, *Escherichia coli*, and *Pseudomonas aeruginosa*) bacteria, and 2 yeasts (*Saccharomyces cerevisiae* and *Cryptococcus neoformans*), representing a diverse range of microorganisms with varying degrees of resistance to cell wall lysis. Three aliquots from nine faecal samples (for a total of 27 samples) were spiked with MOCK.

### 4.3. DNA Extraction Methods

Three aliquots from 16 faecal samples (for a total of 48 samples), including the spiked and MOCK samples (for a total of 78 samples), were independently subjected to DNA extraction with three different protocols (PE-QIA, TAT132H, and T180H) as follows:

QIAmp Fast DNA Stool mini kit modified (PE-QIA): Two hundred mg of stool was washed with phosphate-buffered saline (PBS) (pH 7.4) and centrifuged to remove faecal debris prior to DNA extraction. A pre-extraction step was ad hoc designed to better perform the DNA extraction from both Gram-negative and Gram-positive bacteria. This step consists of mechanical lysis, in which samples were added with glass beads, acid-washed (Sigma Aldrich), and vortexed for 30′ at 300 rpm, and thermal lysis, which consisted of incubation for 30′ at 65 °C and for 30′ at −80 °C. DNA was extracted using a manual column-based DNA extraction method, the QIAmp Fast DNA Stool mini kit, according to the manufacturer’s instructions (QIAGEN GmbH, Hilden, Germany).

Stool DNA and RNA Extraction Kit (T180H): One ml of pre-treatment solution REAG3 was added to a maximum of 200 mg of stool, vortexed, and incubated at 70 °C for 5′. Each sample was centrifuged at 14,000 rpm for 1′. Then, 10 µL of k-proteinase REAG2 was added to 200 µL of sample supernatant and loaded into the Libex Nucleic Acid Extractor, according to the manufacturer’s instructions (Xi’an Tianlong Science and Technology Co., Ltd.-Xi’an, China).

Bacterial Genomic DNA Extraction Kit (TAT132H): One mL of PBS was added to 200 mg of stool, mixed by continuous vortexing, and centrifuged at 500 rpm for 5′ to collect the supernatant. This step was repeated twice. All samples were centrifugated at 5000 rpm for 10’ to collect the pellet. One hundred and eighty microlitres of Lysozyme solution (REAG3) and 20 µL of k-proteinase solution (REAG2) were added to the pellet, mixed, and incubated at 50 °C for at least 30′. Two hundred microlitres of bacterial digestion buffer (REAG5) were added to each sample and mixed before loading into the Libex Nucleic Acid Extractor, according to the manufacturer’s instructions (Xi’an Tianlong Science and Technology Co, Xi’an, China).

The DNA yield (ng) and purity (absorbance ratio at 260/280) of the extracted DNA were determined spectrophotometrically using the NanoDrop^®^ ND-1000 (Nanodrop Technologies Inc., Wilmington, NC, USA), where pure DNA is defined as having a 260/280 absorbance ratio ranging between 1.7 and 2.0.

### 4.4. Bacterial Library Amplification Methods

The bacterial libraries were obtained by amplification of the 16S rRNA V3–V4 regions (~460 bp), using the primers 16S_F 5′-(TCG TCG GCA GCG TCA GAT GTG TAT AAG AGA CAG CCT ACG GGN GGC WGC AG)-3′ and 16S_R 5′-(GTC TCG TGG GCT CGG AGA TGT GTA TAA GAG ACA GGA CTA CHV GGG TAT CTA ATC C)-3′ as described in the MiSeq rRNA Amplicon Sequencing protocol (Illumina, San Diego, CA, USA).

On 14 samples derived from each DNA extraction method, we compared the amplification efficiency of two protocols: the *home brew* protocol [39] and the VeriFi kit (PCR Biosystems simplifying research, London, UK).

*Home brew method:* The first PCR reaction was performed with the following reaction mixture: 1X Buffer, 2.5 mM MgCl_2_, 5 μM of each Primer, 2 mM dNTPs, and 0.5 U Fast Start Hifi Taq (Roche Diagnostics, Mannheim, Germany), with the following conditions: initial denaturation at 95 °C for 3′, 32 cycles of denaturation at 95 °C for 30″, annealing at 55 °C for 30″, extension at 72 °C for 30″, and a final extension step at 72 °C for 5′. DNA amplicons were purified using CleanNGS magnetic beads (CleanNA, Waddinxveen, The Netherlands) and barcoded with unique index combinations using Nextera primers (Illumina, San Diego, CA, USA) and 1X KAPA Hifi HotStart ready Mix (KAPA Biosystems Inc., Wilmington, MA, USA) according to the manufacturer’s protocol, at the same temperature and time conditions as described above, for 12 cycles.

VeriFi Library Amplification Mix for NGS Kit (PCR Biosystems simplifying research, London, UK, named VeriFi Kit)*:* The first PCR reaction was performed using the following reaction mixture: 1X VeriFi™ Hot Start Polymerase, 3 mM MgCl_2_, 1 mM dNTPs (PCR Biosystems simplifying research, London, UK), and 10 µM of each primer mix. PCR amplifications were set up as an initial denaturation at 95 °C for 60″, followed by 35 cycles of denaturation at 95 °C for 15″, annealing at 55 °C for 15″, extension at 72 °C for 30″, and a final extension step at 72 °C for 5′. DNA amplicons were purified using CleanNGS magnetic beads (CleanNA) and barcoded with unique index combinations using Nextera primers (Illumina) and 2X VeriFi™ Library Amplification Mix (PCR Biosystems simplifying research, London, UK) at the same temperature and same time conditions as above described for 12 cycles.

### 4.5. Sequencing

Final libraries were purified using CleanNGS magnetic beads (CleanNA) and quantified using the Quant-iT™ PicoGreen^®^ dsDNA Assay Kit (Thermo Fisher Scientific, Waltham, MA, USA). Bacterial libraries were pooled and diluted to equimolar concentrations (4 nM), then denatured and diluted prior to 5.2 pM sequencing using MiSeq Reagent kit v2 (500 cycles) on an Illumina MiSeq^TM^ platform (Illumina) according to the manufacturer’s specifications to generate 250 base-length paired-end reads [40].

### 4.6. External Contamination Monitoring

The standard operating procedures (SOPs) for 16S rRNA metagenomics include various control checkpoints to monitor and prevent bias resulting from external contamination. Negative controls (no sample) have been included in each experimental session for DNA extraction, DNA amplification, and sequencing to help detect contamination. Furthermore, robust bioinformatic pipelines exclude chimeric sequences generated during the amplification step (Table S1).

### 4.7. Bioinformatic Analyses

Raw data were pre-processed using QIIME2 [41]. Briefly, paired reads were denoised and joined at 99% of similarity using the q2-DADA2 plugin [42] to obtain the amplicon sequence variants (ASVs). Each amplicon was referenced and taxonomically assigned with the q2-Greengenes 2 plugin using the latest version of the Greengenes nucleotide database (v2022.10) [43].

To evaluate the impact of DNA extraction and amplification methods on bacterial community composition, we performed several statistical analyses. Alpha diversity indices, calculated using the Shannon-Weiner and Simpson estimators, were compared across groups with the pairwise Wilcoxon rank test, while beta diversity dissimilarities, computed using the Bray–Curtis algorithm, were assessed with PERMANOVA test. Additionally, the effects of DNA extraction methods on sequencing parameters such as number of reads per sample, ASV frequencies, and number of chimeras were evaluated using the Wilcoxon rank test. Spearman’s correlation analysis, implemented with the corr R function, was employed to assess the relationship between the number of reads and the number of chimeras.

Relative abundances of ASVs, collapsed to phylum and genus levels, were calculated. The top 20 most abundant bacteria were visualized with bar charts to show the mean relative abundance of each sample’ group, and with heatmaps to show the bacterial abundance per sample.

Finally, differential abundance analysis was performed at the genus level using relative frequencies normalized with the cumulative sum scaling (CSS) method [44]. The analysis was conducted with the linear discriminant analysis (LDA) effect size (LEfSe) approach to identify taxa with significant differences between groups [45].

## 5. Conclusions

In conclusion, our study showed that both PE-QIA and T180H achieved similar results in terms of their DNA yield and purity. Furthermore, PE-QIA achieved the best sequencing performance compared to the other two methods and also demonstrated the ability to effectively lyse both Gram-positive and Gram-negative bacterial cells. However, our study showed that T180H is emerging as a viable alternative for time optimisation, especially when dealing with large sample volumes. Furthermore, we have shown that downstream PCR-based steps can interfere with microbiota profiling, but by using an accurate bioinformatic pipeline that minimises the presence of chimeras, the impact on the procedure is minimal.

## Figures and Tables

**Table 1:** DNA yield and purity extracted from faecal, Microbial Community Standard (MOCK), and spiked samples using the three kits.

		PE-QIA		T180H		TAT132H	
		DNA (ng/μL)	260/280 nm	DNA (ng/μL)	260/280 nm	DNA (ng/μL)	260/280 nm
Faecal samples	FB1	47.8	1.98	42.8	1.92	75.7	1.6
	FB2	42.1	1.93	41.1	2.07	22.2	1.49
	FB3	31.8	1.97	32.9	2.14	12.5	1.57
	MB1	2.8	2.06	10.4	1.95	17.6	1.39
	MB2	2.2	1.57	17.1	2.58	8.5	1.66
	MB3	1.7	2.07	11.9	2.16	14.7	1.53
	AC1	3.5	1.8	10.1	2.13	53.8	1.47
	AC2	4.7	1.93	11.8	2.28	26.7	1.47
	AC3	3.7	3	19	2.04	20.7	1.65
	IRC1	7.6	1.77	27.4	2.17	7.8	1.65
	IRC2	4.7	1.66	21.1	2.43	8.4	1.76
	IRC3	5.5	1.88	12.9	2.4	25	1.52
	SE1	1.6	1.28	17.9	2.08	28.3	1.39
	SE2	1.7	4.11	14	2.65	18.5	1.39
	SE3	1.2	2.71	27.8	2.35	35.1	1.42
	NM	1.7	1.03	17.4	2.1	51	1.42
Microbial Community Standard (MOCK)	MOCK-ST	5	2.2	6.3	1.75	11.6	1.56
Spiked samples	FB1-M	18.9	1.96	72	2.05	11.9	1.57
	FB2-M	28.4	1.97	83	2.03	16.5	1.41
	MB2-M	2.9	2.33	7.2	1.66	9.8	1.51
	AC1-M	5.2	2.95	16.3	2.05	18.9	1.26
	AC2-M	4.2	2.62	17.6	2.12	4.8	1.93
	IRC1-M	3.1	2.09	22.6	2.21	6	2.57
	IRC2-M	3.8	2.3	24.3	1.88	24.7	1.81
	SE1-M	2.5	2.8	27.3	2.21	13	1.57

**Table 2:** Bacterial genera filtered for statistically significant *p*-values by the LEfSe analysis and grouped according to their Gram classification.

Direction	Taxa	GRAM
PE-QIA	*Bacteroides*_H	-
	*Dysosmobacter*	-
	*Butyricimonas*	-
	*Parabacteroides*_B_862066	-
	*Phocea*	-
	*Lawsonibacter*	+
	*Enterocloster*	+
	*Intestinimonas*	+
T180H	*Phocaeicola*_A_858004	-
	*Alistipes*_A_871400	-
	*Sphingomonas*_L_486704	-
	*Odoribacter*_865974	-
	*Veillonella*_A	-
TAT132H	*Gemmiger*_A_73129	-
	*Pauljensenia*	+
	*Collinsella*	+
	*Streptococcus*	+
	*Eggerthella*	+
	*Bifidobacterium*_388775	+
	*Dorea*_A	+
	*CAG-1427*	+
	*Lactobacillus*	+
	*Blautia*_A_141781	+
	*Anaerofustis*	+
	*Gordonibacter*	+
	*Blautia*_A_141780	+
	*Lachnoclostridium*_B	+
	*Copromorpha*	+
	*Marvinbryantia*	+
	*Faecalicoccus*	+
	*Scatomonas*	-

**Table 3:** Table of bacterial taxa identified by sequencing selected on the basis of MOCK theoretical composition.

	Bacterial Taxa ^1^	*Bacillus* P_294101	*Enterococcus* H_360604	*Escherichia* 710834	*Limosilactobacillus*	*Listeria*_A	*Pseudomonas* E_650326	*Salmonella* 692099	*Staphylococcus*	% ^3^
	Theoretical composition ^2^	17.4	9.9	10.1	18.4	14.1	4.2	10.4	15.5	-
MOCK	T180H	10.3	7.27	18.26	36.47	0.14	20.72	0	3.76	87.5%
	PE-QIA	13.99	11.77	28.8	19.55	0	19.09	0	2.49	75%
	TAT132H	9.44	3.15	5.02	2.72	0	2.7	0	0.59	75%
Faecal spiked samples	T180H	7.19	4.56	17.55	19.91	0	11.89	0.11	1.16	-
	PE-QIA	0.73	0.81	4.63	1.39	0.04	0.84	0	0.63	-
	TAT132H	3.47	1.08	1.78	0.83	0.01	0.82	0	0.87	-

^1^ Reported as Bacillus subtilis, Enterococcus faecalis, Escherichia coli, Lactobacillus fermentum, Listeria monocytogenes, Pseudomonas aeruginosa, Salmonella enterica, and Staphylococcus aureus in the data sheet of ZymoBIOMICS Microbial Community Standard; ^2^ Theoretical composition in % reported in the data sheet of ZymoBIOMICS Microbial Community Standard; ^3^ The percentage of correctly identified bacteria out of the total number of expected bacteria is shown.

## Data Availability

The raw data supporting the conclusions of this article will be made available by the authors on request.

## Supplementary Materials

The following supporting information can be downloaded at: https://www.mdpi.com/article/10.3390/ijms26115226/s1.
